# Experimental, Theoretical and Simulation Studies on the Thermal Behavior of PLA-Based Nanocomposites Reinforced with Different Carbonaceous Fillers

**DOI:** 10.3390/nano11061511

**Published:** 2021-06-07

**Authors:** Giovanni Spinelli, Rosella Guarini, Rumiana Kotsilkova, Evgeni Ivanov, Vittorio Romano

**Affiliations:** 1 Open Laboratory on Experimental Micro and Nano Mechanics (OLEM), Institute of Mechanics, Bulgarian Academy of Sciences, Acad. G. Bonchev Str. Block 4, 1113 Sofia, Bulgaria; rgrosagi@gmail.com (R.G.); kotsilkova@yahoo.com (R.K.); ivanov_evgeni@yahoo.com (E.I.); 2 Research and Development of Nanomaterials and Nanotechnologies (NanoTech Lab Ltd.), Acad. G. Bonchev Str. Block 4, 1113 Sofia, Bulgaria; 3 Department of Industrial Engineering, University of Salerno, Via Giovanni Paolo II, 132, 84084 Fisciano, Italy; vromano@unisa.it

**Keywords:** biodegradable polymers, graphene, carbon nanotubes, nanocomposites, thermal transport properties, design of experiments, multiphysics simulations

## Abstract

Many research efforts have been directed towards enhancing the thermal properties of polymers, since they are classically regarded as thermal insulators. To this end, the present study focuses on the thermal investigation of poly(lactic acid) (PLA) filled with two types of carbon nanotubes (trade names: TNIMH4 and N7000), two type of graphene nanoplatelets (trade names: TNIGNP and TNGNP), or their appropriate combination. A significant increase in the thermal conductivity by 254% with respect to that of unfilled polymer was achieved in the best case by using 9 wt% TNIGNP, resulting from its favorable arrangement and the lower thermal boundary resistance between the two phases, matrix and filler. To theoretically assist the design of such advanced nanocomposites, Design of Experiments (DoE) and Response Surface Method (RSM) were employed, respectively, to obtain information on the conditioning effect of each filler loading on the thermal conductivity and to find an analytical relationship between them. The numerical results were compared with the experimental data in order to confirm the reliability of the prediction. Finally, a simulation study was carried out with Comsol Multiphysics^®^ for a comparative study between two heat sinks based on pure PLA, and to determine the best thermally performing nanocomposite with a view towards potential use in heat transfer applications.

## 1. Introduction

Nowadays, the high demand for custom-made products requires the development of new materials. Polymers have been identified as promising candidates for this aim, to the point where their current impact on our lives is almost unquantifiable. In fact, polymer-based products have been favorably adopted everywhere: synthetic fibers are also increasingly being used for clothing production, plastic bags are adopted for multiple, epoxy glues (and not only them) are widely present in the field of adhesives, fiberglass or carbon-based reinforced composites are being used as structural parts, and so on, with the list being potentially endless [1]. Noteworthy is the recent study on the possibility of using fibers obtained from polyethylene terephthalate (PET) waste bottles to improve the ductility of concrete [2], as well as the efforts in selecting appropriate nanoclays to combine with polyamide (PA) fibers in order to improve the flame-retardant and tensile properties of knitted fabrics [3]. Due to the increasing interest in environmentally friendly materials over the years, poly(lactic acid) (PLA) has aroused a great deal of attention, and therefore it is being intensely investigated, in both industry and academia [4,5]. The creation of non-woven membrane supports made with bamboo-fiber-reinforced poly(lactic acid) composites for an energy-efficient and sustainable technology is of interest [6], as well as the design of poly(lactic) acid bio-composites that include three types of silk fibers and wool protein microparticles for targeted biomedical applications [7]. More recently, with the advent of new fabrication processes based on additive manufacturing (AM) technologies, PLA, as a result of its vegetable-based nature, which confers excellent biocompatibility, sustainability and biodegradability, has become increasingly widespread, to the extent that it represents the primary natural raw material used in extrusion-based 3D printing techniques (popularly known as fused deposition modeling, FDM) [8]. This technology relies on a continuous filament consisting of a thermoplastic polymer that is fed by the extrusion head to the nozzle, where it melts, at a selected temperature, to then subsequently re-solidify on a building plate, thus creating, layer-by-layer in a pre-determined path, the designed 3D object [9,10]. Compared to classical fabrication processes with a subtractive nature, additive manufacturing technology presents remarkable benefits in terms of cost effectiveness, reduced processing waste, light weight, and versatility in the manufacturing of complex structures, which are increasingly being used in several fields [11]. To cite just some of them, AM has been adopted as a fast prototyping technology in the early stages of the design and development of components in aerospace and defense fields [12], for the manufacture of biomaterials suitable in medical applications and in dental care [13,14], and to produce polymer-based packages with electromagnetic (EM) shielding properties or heat exchangers, which are essential in the electronic industry [15]. Nevertheless, despite all of the progress made so far in terms of the development of AM techniques, the selection of suitable materials remains a critical bottleneck for their full implementation [16,17]. Thermal dissipation and electrical insulation are challenging issues that have still not been overcome when it comes to polymer-based composites. Therefore, different research efforts have been devoted to their improvement in terms of both thermal and electrical conductivity [18,19,20,21,22]. The negligible values of thermal conductivity typical for polymers are due to their structures; the random structure of molecule chains strongly limits the thermal transport due to the phonons, and as result, low values of thermal conductivity are observed at the macroscale level. Although the thermal conductivity can be affected by different intrinsic features of polymers, to engineer the polymeric matrix through the dispersion of highly conductive fillers inside it, it is recognized as a valid method for enhancing the overall thermal performance of the resulting materials, regardless of the thermoplastic or thermosetting nature of the host polymer [23]. Table 1 summarizes the thermal conductivity values of some polymers commonly used in the AM field, as well as for some classic fillers, which are grouped according to their physical type, i.e., carbon-based, metallic, and ceramic fillers [24]. Information on other polymers is reported in the handbook written by Yang [25].

On the basis of the analysis of these data, it is evident that the thermal conductivity of the fillers, which is always dependent on the material structure, is significantly higher than that of the neat polymers.

Heat transfer can be associated with purely phonon mechanisms, or with the combined effect of phonons and electrons. Therefore, carbon-based and metallic fillers, the reticular structures of which contain freely moving electrons, present a dual heat conduction mechanism that leads to higher thermal conductivity compared to the values exhibited by ceramic fillers which are characterized only by a phonon transmission [26]. Carbon-based fillers (used either as single fillers or in combination) are considered ideal candidates for obtaining advanced composites, not only due to their remarkable thermal and electrical conductivities, but also due to their extraordinary corrosion resistance and thermal expansion coefficients, which are lower than other reinforcement particles. A scientifically recognized theory explaining both the thermal and the electrical conduction in composite structures is the so-called percolation theory, which is based on the creation of suitable electrically/thermally conductive paths: as soon as the filler concentration reaches a suitable value (percolation threshold), continuous paths (network) will be established, and the heat/current can flow through them due to the lower thermal/electrical resistance pathways, which are otherwise not present in the host polymers. As a result, a significant improvement in the thermal and electrical conductivity is observed [27]. 3D-printing silicone acrylate-based formulations (Polydimethylsiloxane, PDMS) with enhanced thermal conductivity due to the introduction of boron nitride (BN) as conductive filler were proposed and investigated by Pezzana et al. [28]. A 3D-printed composite with alumina and oriented carbon nanofibers (CFs) with enhanced thermal conductivity compare to that of cast composites was discussed by Ji et al. [29]. PLA-based nanocomposites filled with four different metal particles were prepared by 3D-printing and then characterized in order to investigate the influence of the additive microstructures, along with the printing parameter settings, on the thermal conductivity of the resulting structures. [30]. Even though the properties of the polymers were significantly improved due to the addition of various conductive fillers, a comprehensive knowledge of the overall performance has still not yet been fully achieved, since the observed results are usually far from those theoretically expected. In fact, many literature studies indicate that several factors, including the intrinsic features of filler particles, such as their geometrical shape and aspect ratio (AR), their concentration, their spatial dispersion in the polymer matrix, and the adhesion and interaction at the interface between the constituent phases, strongly affect the thermal and electrical behavior (among other things) of the composites [20,31]. In a previous study [32], the authors investigated formulations based exclusively on a single reinforcement type, MWCNTs or GNPs, in order to analyze the influence of their different geometrical features on the final performance of the resulting materials. Here, the investigation is enriched with the additional analysis of hybrid systems (multiphase nanocomposites), including both types of fillers (MWCNTs/GNPs), in some selected weight ratios, and up to a maximum of 9% total infill weight. Another novelty is represented by the Design of Experiments (DoE), which was performed to identify the most influential design parameters, and the Response Surface Method (RSM), which was applied to find the analytic relationship between them and the parameter of interest, i.e., thermal conductivity. Moreover, simulation studies carried out with the commercial software package Multiphysics (COMSOL Multiphysics^®^) are presented with the aim of predicting the thermal behavior of this novel nanocomposite, designed as a heat sink for potential heat transfer applications.

## 2. Materials and Methods

### 2.1. Materials

The base polymer used in this study for the compounding formulation was Ingeo™ Biopolymer PLA-3D850 (Nature Works, Minnetonka, MN, USA), which is particularly indicated for the manufacturing of 3D printer monofilament, since it is characterized by fast crystallization rate, good adhesion to the build plate, and high printing speed, as well as less warping or curling, low odor emission, and much more. Among its main physical properties, it is worth mentioning its glass transition temperature (*Tg*) of 55–60 °C and peak melt temperature of 165–180 °C (both measured in agreement with the D3418 standard of the American Society for Testing and Materials, ASTM), as well as its melt mass-flow rate (*MFR*) of 7–9 g/10 min (according to the D1238 ASTM standard). The aim was to develop a non-conventional material for additive manufacturing and to obtain, thanks to the potentialities of this technology, heat sinks with an ad hoc design for heat transfer applications.

Four types of carbon nanofiller were chosen to manufacture the nanocomposites investigated in the present study. In brief: (i) industrial graphene TNIGNP (from Times Nano, Chengdu, China); (ii) industrial MWCNTs—TNIMH4 (from Times Nano, Chengdu, China); (iii) graphene—TNGNP (from Times Nano, Chengdu, China); and (iv) MWCNTs—N7000 (NC7000™ series, Nanocyl^®^ SA, Sambreville, Belgium).

The names used—(i) industrial graphene nanoplatelets (TNIGNP), and (ii) industrial multiwall carbon nanotubes (TNIMH4)—were provided by the producer (Times Nano, Chengdu, China). The term industrial indicates that their production was carried out in large quantities and at a low price in contrast to higher-quality and more expensive nanofillers, which are produced in small quantities under laboratory or semi-industrial conditions (e.g., TNGNP and N7000). In this study, both industrial fillers and higher-quality fillers were used to compare the characteristics, properties, advantages and disadvantages of the polymeric nanocomposite materials obtained from them.

**Figure 1:**
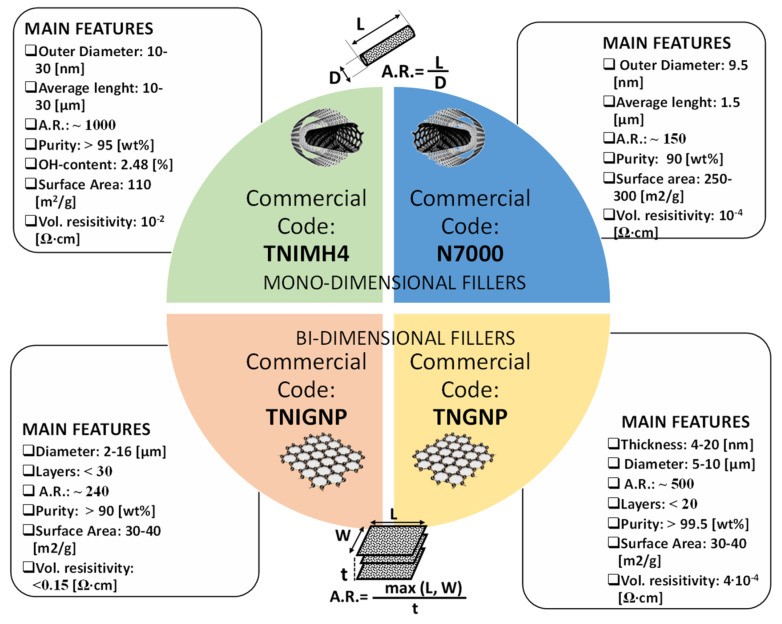
Trade names and technical specifications of each of the fillers used in this study. Source: technical data sheets available on the website of the manufacturing companies.

In particular, the two types of graphene nanoplatelets (GNPs) and the two grades of multiwall carbon nanotubes (MWCNTs) were selected here based on their low price versus good technical specifications. The size, shape, aspect ratio, specific surface area and functionalization of nanoparticles were varied in order to estimate the essential nanofiller characteristics governing the thermal, electrical and other physical properties of the nanocomposites [32]. MWCNTs differ mainly with respect to aspect ratio (1000 for TNIMH4 and 150 for N7000), OH content (2.48% for TNIMH4 and absent for N7000), and surface area (110 m^2^/g and 250 m^2^/g for TNHIMH4 and N7000, respectively). Instead, GNPs differ principally with respect to aspect ratio (240 for TNIGNP and about 500 for TNGNP), volume resistivity (˂0.15 Ω∙cm for TNIGNP and about 10^−4^ for N7000), and purity (˃90% and ˃99.5% for TNIGNP and TNGNP, respectively). Other technical characteristics are summarized in the schematic representation presented in Figure 1.

The definition of the aspect ratio (i.e., A.R.) for both 1-dimensional filler (MWCNTs) and 2-dimensional ones (GNPs) is also schematically reported in Figure 1. The aspect ratio of an object is defined as the ratio of its longest dimension to its shortest one. For rodlike fillers such as carbon nanotubes or nanofibers, the A.R. coincides with the ratio of the length (*L*) to the diameter *D* (*A.R.* = *L/D*), whereas for planar particles, such as graphene and its derivatives, it is given by the ratio of the largest lateral dimension (length, *L* or width, *W*) to the thickness *t* (*A.R.* = *max (L, W)/t*).

### 2.2. Preparation of Nanocomposites and Test Samples

Two sets of nanocomposites were prepared by varying the type and content of the fillers (1.5–9 wt%). The first set was prepared by melt mixing industrial graphene (TNIGNP) and industrial multi-walled carbon nanotubes (TNIMH4) with PLA 3D850 in pellet form. The second set of nanocomposites was obtained using the same host matrix (PLA 3D850) milled to a powder but filled with the other types of graphene nanoplatelets (TNGNP) and MWCNTs (N7000). The mono (PLA/MWCNT and PLA/GNP) and bi-filler nanocomposites (PLA/MWCNT/GNP) were processed by melt extrusion at 170–180 °C using a twin-screw extruder (COLLIN Teach-Line ZK25T, Maitenbeth, Germany), at a screw speed of 40 rpm. The PLA and nanofillers were dried for 4 h at 80 °C in a vacuum oven, and masterbatches of 9 wt% filler were initially extruded, and then further diluted with PLA by a second extrusion run to produce mono-filler composites with varying filler contents. The bi-filler composites with 3, 6 and 9 wt% total filler content and various GNP: MWCNT ratios were fabricated by mixing the GNP/PLA and the MWCNT/PLA masterbatches with the neat PLA in appropriate amounts.

The concentrations were identified during the pre-planning phase in order to perform a Design of Experiment (see Section 2.3.4. Design of Experiments (DoE) for Thermal Characterization), initially based on uniformly distributed parameters at four levels in the interval [0÷9] wt%, and eventually with further intermediate values being considered in a second stage, if necessary. The maximum amount of 9 wt% was dictated by a compromise in terms of overall the performances exhibited by the resulting nanocomposites and their easier processability, on the basis of our previous study [32].

Table 2 summarizes the compositions studied in the present study. Circular-shaped specimens with a diameter of 16 mm and a thickness of 3 mm were hot pressed from extruded pellets, polished and then used for electrical and thermal conductivity tests.

### 2.3. Experimental and Numerical Methods

#### 2.3.1. Morphological Analysis

The dispersion state of the nanofillers inside the PLA-based nanocomposites, as well as their morphological features, was investigated by means of transmission electron microscopy (TEM) and scanning electronic microscopy (SEM). More specifically, the transmission electron microscopy (TEM) analysis was carried out using an FEI TECNAI G12 Spirit-Twin (LaB6 source, FEI Company, Hillsboro, OR, USA) working with an acceleration voltage of 120 kV with a magnification variable between 22 and 300 kX in combination with an FEI Eagle-4k charged coupled device camera (CCD). Prior to the investigation, sections of the specimens suitable for analysis were cut, at room temperature, using using a Leica EM UC6/FC6 ultramicrotome, and they were then positioned on 400 mesh TEM copper grids. With respect to the SEM, a JSM-6700F apparatus (JSM-6700F, Jeol, Akishima, Japan) was used on suitable fragments of the whole specimens obtained through fragile fractures in liquid nitrogen. Prior to the investigation, the samples were chemically etched and then gold-sputtered following a method already described by Spinelli et al. [33,34], and therefore omitted here.

#### 2.3.2. DC Electrical Conductivity Analysis

**Figure 2:**
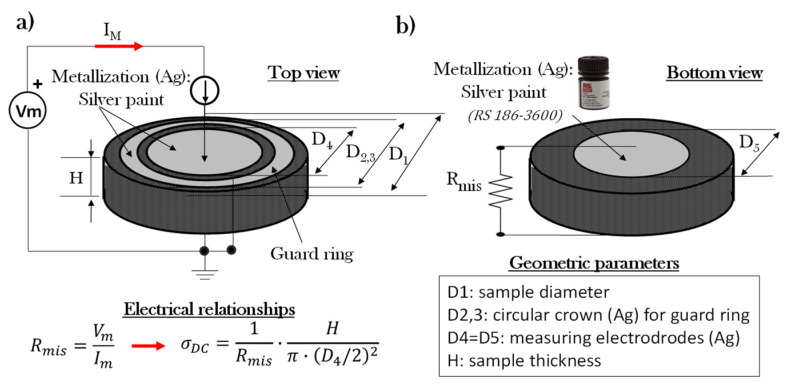
Setup for the measurement of the DC bulk conductivity of circular-shaped specimens with top view in (**a**) and bottom view in (**b**), respectively. Geometric details and the electrical relationships adopted for its determination are also reported.

The bulk conductivity (at room temperature of 20 °C) of each formulated composition was tested using a pico-ammeter (Keithley 2400, Keithley Instruments Inc., Beaverton, OR, USA), which acted simultaneously as both a source and a meter, in accordance with the electrical schematization reported in Figure 2a). Three samples were measured for each nanocomposite, and the average values of the measurements are reported as results in the electrical section of the present study. During the test, the electrical resistance (*R_mis_*) of the material was measured by applying Ohm’s first law between the DC voltage applied to the samples (*Vm*) and the measured current (*Im*) flowing in it (*R_mis_* = *Vm/Im*). The electrical conductivity, *σ_DC_* [S/m], of the bulk sample was determined using Ohm’s second law:

(1) $$\sigma_{DC}=\frac{1}{R_{mis}}\cdot \frac{H}{\pi \cdot {\left(D_{4}/2\right)}^{2}}$$

where *H* is the sample thickness and *D*_4_ is the sample diameter of the metalized measuring electrodes. In fact, in order to ensure Ohmic contacts, both sample surfaces, see Figure 2a,b, were covered with a silver paint having a volume resistivity of 0.001 Ω·cm (Alpha Silver Coated Copper Compound Screening, RS 186–3600, Corby, UK). Moreover, in order to guarantee the exclusive measurement of bulk currents, a guard ring was applied on the top side of the sample in order to drain any surface current towards the mass of the system, especially for composites with high electrical conductivity.

#### 2.3.3. Thermal Characterization

**Figure 3:**
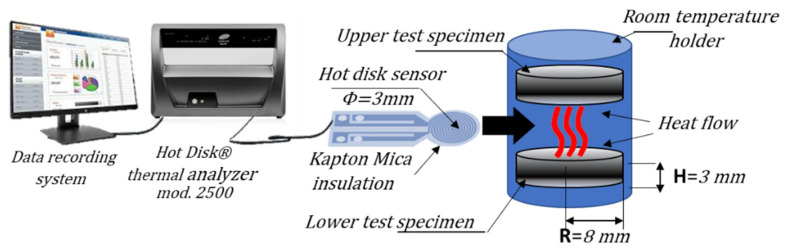
Experimental setup for the thermal characterization performed by using a Hot Disk^®^ thermal constant analyzer.

A Hot Disk 2500 thermal constant analyzer (Hot-Disk AB TPS 2500, Göteborg, Sweden) was used to measure the thermal conductivity of the GNP- and MWCNT-based polymer composites using the transient plane source method (i.e., TPS) in accordance with the specifications of ISO 22007-2-2015 (International Organization for Standardization) [35]. Before measurements, the samples were polished using sandpaper to obtain very flat surfaces. The measurements were performed by placing the TPS element (3 mm diameter), which acts simultaneously as a heater and a temperature sensor, between two similar slabs of material (Figure 3). The sensor supplied a heat pulse of 0.01 W for 40 s to the sample at room temperature, and the associated change in temperature was recorded. The thermal conductive parameters, including the thermal conductivity and the thermal diffusivity of the samples, were measured according to the theory already described in detail in Spinelli et al. [30], and only briefly summarized here.

The experimental measurements were correlated with the time-dependent resistance of the TPS element, and were evaluated in accordance with the following expression:

(2) $$R\left(t\right)=R_{0}\left[1+\beta \Delta T\left(\tau \right)\right].$$

In Equation (2), above, *R*_0_ represent the initial value for the resistance of the TPS before the transient recording (about 4 Ω at room temperature), and β is its temperature coefficient, whereas ΔT(τ) is the temperature increase recorded over time, calculable by means of the formula:

(3) $$\Delta T\left(\tau \right)=\frac{P_{0}}{\left(\pi^{3/2}r\lambda \right)}D\left(\tau \right)$$

where the dimensionless time $\tau ={\left(t\cdot \alpha /r^{2}\right)}^{1/2}$ is dependent on the measurement time *t*, the thermal diffusivity α, the radius of the sensor *r*, the input heating power *P*_0_, and the thermal conductivity *λ*. Finally, D(τ) is a Bessel-based dimensionless shape function that also accounts for the number of concentric circles forming the hot disk sensor [34].

Once the temperature evolution has been determined by means of Equation (2) and by fitting it to Equation (3), it is possible to derive the thermal features of the material under test.

#### 2.3.4. Design of Experiments (DoE) for Thermal Characterization

Design of Experiments (DOE) belongs to the applied statistics branch introduced for evaluating so-called cause-effects, i.e., the relationship between the factors conditioning a process/product and its output or performance function (*PF*) of interest [36,37].

**Figure 4:**
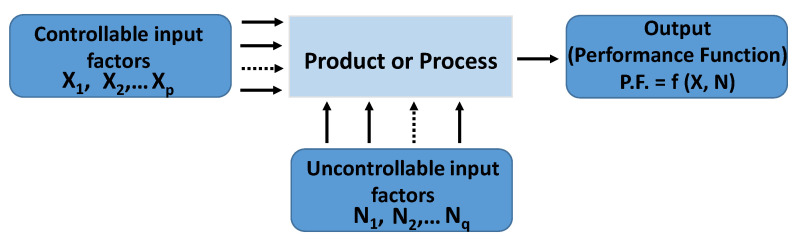
Schematic illustration of a system (product or process) at the design stage.

As schematically shown in Figure 4, during the initial pre-planning design phase, the most common elements to be considered include the controllable input factors (i.e., *X_i_*, which can be arbitrarily modified), the uncontrollable input factors (i.e., *N_i_*, which cannot be changed due to noise sources and obvious tolerances on *Xi* variables), and the output of the process (the performance function of interest, *P.F.*) The latter is the result of the joint action of controllable input factors (X = (X_1_, X_2_, …, X_p_)) and uncontrollable input factors (N = (N_1_, N_2_, …, N_q_)), i.e., *P.F.* = f (X, N). In the presence of multiple design parameters, DoE is a powerful statistical tool that makes it possible to identify the most influential one among them. Moreover, DoE helps to choose the best combination of them for optimizing the selected *P.F.*, or at least to contain its deviation in response to the action of uncontrollable factors (robust design, RD) [38].

An approach based on DoE was successfully adopted to produce polymer microfibers with carbon nanotubes through electrospinning experiments [39], and has recently been combined with artificial intelligence (AI) to develop the sustainable electrochemical synthesis of zeolitic imidazolate frameworks (ZIF-8) [40].

In this paper, DoE is performed to analyze the influence of the two main controllable input factors, i.e., the nanofiller loading ($wt{\%}_{MWCNTs},wt{\%}_{GNPs}$) on the thermal conductivity (i.e., λ) of the resulting nanocomposites, which represents the targeted performance function in this analysis. For this purpose, a specific Matlab^®^ routine was designed. As a matter of course, a discretization level for the controllable input factors should be properly set. Uniformly distributed parameter values are advisable at first, whereas further intermediate points may be considered in order to refine the model at a later stage [41]. In any case, in order to obtain an effective predictive model, a good space-filling of the experimental region data must be ensured. In light of the above, in the present study, the input variable vector was:

(4) $$\bar{x}=(wt{\%}_{MWCNTs},wt{\%}_{GNPs})\;\epsilon \;{\mathbb{R}}^{2}$$

where a discretization over four levels was selected for the two controllable input factors in the interval [0÷9] wt%, i.e.,:

(5) $$wt{\%}_{MWCNTs\_1}=0,wt{\%}_{MWCNTs\_2}=3,\;wt{\%}_{MWCNTs\_3}=6,\;wt{\%}_{MWCNTs\_4}=9\;$$

and:

(6) $$wt{\%}_{GNPs\_1}=0,wt{\%}_{GNPs\_2}=3,\;wt{\%}_{GNPs\_3}=6,\;wt{\%}_{CNPs\_4}=9\;$$

As result, the variable space is compact:

(7) $$D=wt{\%}_{MWCNTs}\times wt{\%}_{GNPs}\subset {ℜ}^{2}$$

whereas the *P.F*. is estimated for each ordered pair $\left(x_{1},x_{2}\right)$ of the controllable input factors, i.e.,:

(8) $$\left(x_{1},x_{2}\right)=(wt{\%}_{MWCNTs},wt{\%}_{GNPs})\epsilon D.$$

By following the aforementioned methodological steps, DoE on the basis of 2^4^ = 16 points ϵ D*⊂ *D* generates scattered data for the *P.F.*, which is required for carrying out sensitivity analysis by means of Dex Scatter Plot (DSP), Main Factor Plot (MFP) and Response Surface Methodology (RSM). Their meaning, along with their relative results, will be illustrated in the corresponding Results sections.

#### 2.3.5. Multiphysics Simulations of Thermal Properties

**Figure 5:**
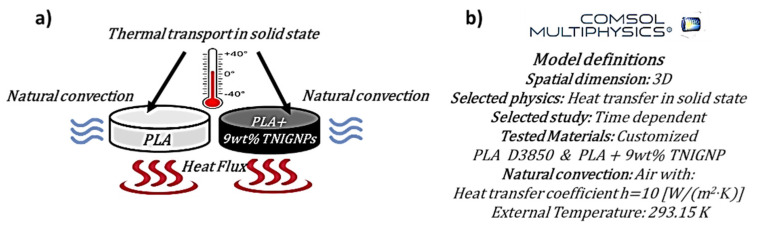
Schematic illustration (**a**) of the simulation studies carried out on pure PLA and on the best (in terms of thermal properties) composite (PLA + 9 wt% TNIGNPs). The main model definitions adopted in COMSOL are also reported (**b**).

In the present work, we study the thermal behavior of designed nanocomposites in the case of their use as thermal dissipators in heat transfer applications. The considered samples, modeled as cylinders with a diameter of 16 mm and a height of 3 mm, include one of pure polymer (PLA), with the others being the best-performing nanocomposites, in terms of thermal properties, investigated here, i.e., PLA including 9% by weight of TNIGNP (as schematically shown in Figure 5a). For this purpose, a mathematical model was developed, and a numerical analysis based on the Finite Element Method (FEM) was performed using the commercial software COMSOL Multiphysics^®^. The main model definitions selected for the numerical analysis are summarized in Figure 5b.

**Figure 6:**
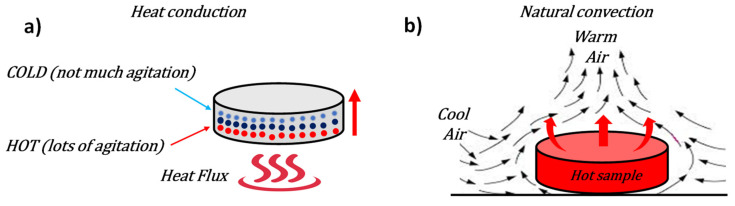
Schematic illustration of the two main modes of thermal energy transfer: (**a**) heat conduction and (**b**) natural convection.

With respect to potential applications as heat sinks, the heating of the sample was simulated by applying a heat flux (900 W/m^2^) to the bottom surface and then cooling in still air at a temperature of 293.15 K. The heat is transferred from the hot surface to the inside the sample by conduction (Figure 6a) and to the surrounding air by natural convection, a consequence of the different density between the hot air close to the heated surface and the cold air surrounding the lateral surface and the upper surface of the sample (Figure 6b).

The mathematical theory of heat conduction was formulated by Fourier, and the basic equation (in differential form), bearing his name, governs conduction heat transfer in accordance with the following vectorial equation:

(9) q=−λ·∇T

where the involved parameters are as follows (including the SI unit):

- *q* is the heat flux, i.e., the rate of heat flow per unit area [W/m^2^];
- *λ* is the intrinsic thermal conductivity of the material, assumed to be constant in the present work [W/m∙K];
- ∇T is the temperature gradient [K/m].

In several simple applications, Fourier’s law can be expressed in one-dimensional form in a generic i direction, in which case Equation (9) can be written as:

(10) $$q_{i}=-\lambda \cdot \frac{\mathrm{dT}}{di}\;with\;i=x,y\;or\;z\;axis$$

It should be noted that the algebraic sign reported in Equation (9) and in Equation (10) is used when the heat flux occurs in the opposite direction to the temperature gradient, as occurs in the case in question, in which the temperature decreases in the direction in which the radius increases. In the case of interest for the temperature profile inside the medium, the mathematical formulation, considering both the law of energy conservation and Fourier, leads to the universally known differential equation for heat conduction for a solid at constant pressure, written in vectorial form:

(11) $$\nabla \cdot \left(\lambda \nabla T\right)+\dot{q}=\rho \cdot c_{p}\cdot \frac{\partial T}{\partial t}$$

where:

- ρ is the density of the material [kg/m^3^];
- *c_p_* is the specific heat of material [J/kg∙K];
- $\dot{q}$ is the heat generated per unit volume [W/m^3^].

Adapted to the study of our specific nanocomposite solids, the thermal energy Equation (11) can be written in cylindrical coordinates for a differential volume 2π*r*Δ*r*Δ*z*, as [42]:(12)
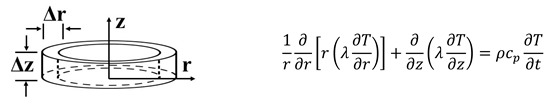



As initial conditions, the material is assumed to be at a uniform temperature T_0_; as boundary conditions, symmetry on the axis for *r* = 0 is assumed, whereas a loss of heat because of natural convection is considered at the external boundaries (*r* = *R*) and at the top surface (*z* = 3 mm); finally, a heat flux is imposed at the bottom surface (*z* = 0), as summarized in Table 3.

The heat transfer coefficient by natural convection *h* [W/m^2^∙K] is defined by Newton’s law of cooling:

(13) Q=h·S·ΔT

it represents the proportionality factor between the heat flow *Q* [W] and the Δ*T* [K], which causes the convective transport between a hot solid surface *S* [m^2^] and the surrounding air.

## 3. Results

### 3.1. Morphological Analysis

**Figure 7:**
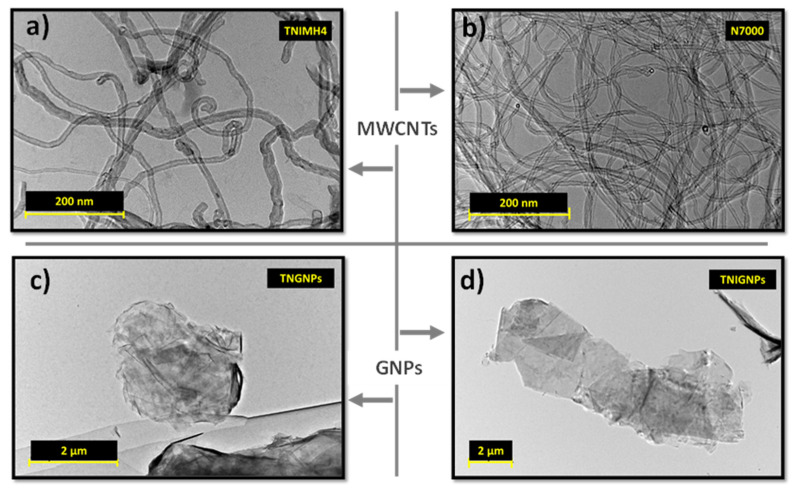
TEM images showing fillers powder of multiwalled carbon nanotubes of the types (**a**) TNIMH4 and (**b**) N7000, and graphene nanoplatelets of the types (**c**) TNGNPs and (**d**) TNIGNPs, respectively.

Given their crucial roles, it is of particular importance to investigate the basic morphological features of carbon nanotubes and graphene, as well as their dispersion state (also when combined together at low loadings of both fillers) before presenting the electrical and thermal performance of the resulting nanocomposites in which they have been dispersed. Representative TEM images of all four types of carbonaceous fillers (in powder form) used in the present study are shown in Figure 7 with a magnification of 200 nm for MWCNTs and 2 µm for GNPs, respectively.

**Figure 8:**
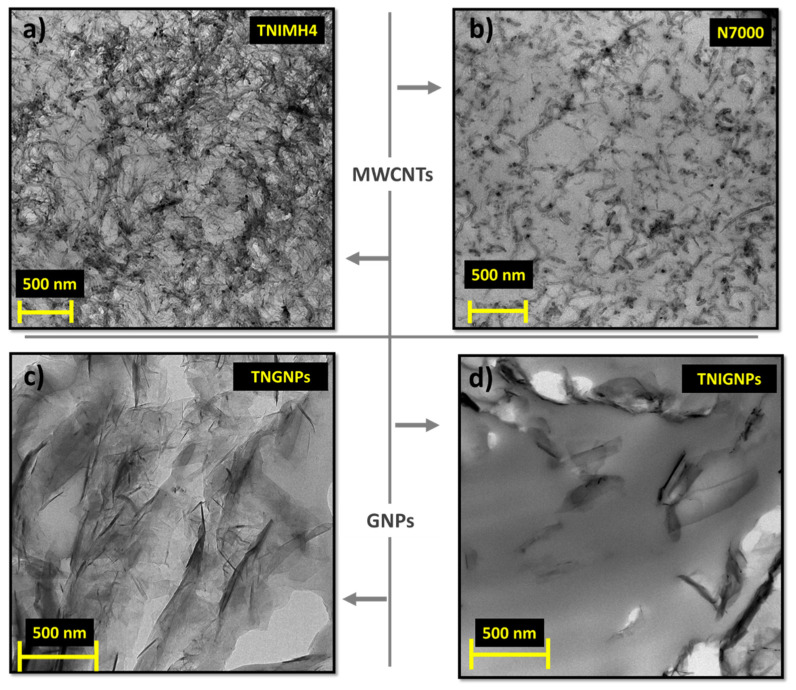
TEM images showing multiwalled carbon nanotubes of the types (**a**) TNIMH4 and (**b**) N7000, and graphene nanoplatelets of the types (**c**) TNIGNPs and (**d**) TNGNPs, respectively, dispersed in the polymer with 9 wt% loading.

On the basis of their quick analysis, it is worth noting the nanotube waviness and the larger size of TNIMH4 with respect to N7000 in terms of both average length and diameter, as well as the relevant dimension of planar fillers (GNPs) compared to the monodimenisonal ones (MWCTNs). Figure 8 presents the TEM images of the aforementioned fillers when incorporated at the maximum investigated loading (9 wt%) in the polymer matrix.

**Figure 9:**
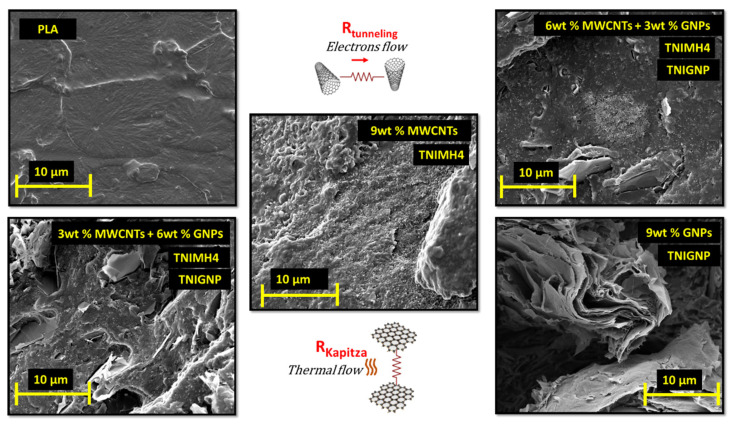
SEM images of pure PLA and different nanocomposites with a total charge of 9 wt% including hybrid systems made with both fillers (TNIMH4 and TNIGNP) in some selected concentrations.

It is noticeable at first sight that the issue of agglomeration between the fillers is more evident for MWCNTs of type TNIMH4 and GNPs of type TNGNP, whereas N7000 and TNIGNP particles appear well dispersed, since the aggregates were limited both in terms of number and dimension. The phenomena of bundles or stable aggregates may occur due to the remarkable intermolecular interaction between the fillers. Consequently, in addition to a non-uniform dispersion, the agglomeration effect reduces the aspect ratio of the nanofillers, which in turn affects the overall properties of final nanocomposites by worsening the performance compared to that expected [43]. Finally, Figure 9 illustrates some selected SEM images with respect to the unfilled PLA and nanocomposites with a total charge of 9 wt% including some hybrid systems based on the simultaneous combination of GNPs and MWCNTs in the selected weight ratio.

A good adhesion and interaction at the matrix/filler interface is observed, since no significant porosity can be identified; only some unavoidable cavities in the case of graphene-based composites. Instead, what is important to point out is that the uniform and homogenous arrangement of carbon nanotubes seems to be more favorable for the creation of the percolating network within the polymer, and therefore, MWCNTs appear to be better indicated for improving the electrical performance of the resulting nanocomposites given the improved electron tunneling between the conductive particles. In contrast, the clearly visible stacked arrangement of the graphene nanoplatelets is certainly suited for a more highly efficient phononic heat flow compared to the nanotubes, due to a lowering of Kapitza resistance, i.e., the interfacial thermal resistance between the two phases, GNPs/matrix. This is because the bi-dimensional surfaces of GNPs are more easily wetted by the polymer. Hybrid systems represent a recent design attempt to achieve a suitable balance in terms of both the electrical and thermal properties of nanocomposites.

### 3.2. DC Electrical Conductivity

**Figure 10:**
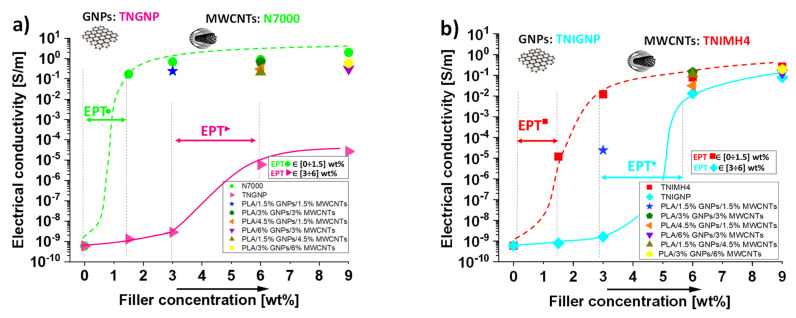
(**a**) DC electrical conductivity of PLA reinforced with different types of carbon-based fillers: TNGNP or N7000 and their combination (**a**) and TNIGNP or TNIMH4 and their combination (**b**).

The addition of conductive nanofillers inside the polymers conditions a whole series of physical properties, including the thermal and electrical ones. Changes in the DC electrical conductivity (*σ_DC_*) of the composites based on PLA/MWCNTs (both types, TNIMH4 and N7000) and PLA/GNPs (both types, TNIGNP and TNGNP) as a function of the filler loading are reported in Figure 10a,b, respectively. The progressive introduction of MWCNTs and GNPs nanofillers determines, especially at higher filler loadings, a remarkable improvement in the electrical conductivity of the nanocomposites with respect to the value exhibited by the pure PLA (5.9 × 10^−10^ S/m). In the best case (i.e., 9 wt% N7000), a conductivity value of about 2 S/m was obtained. For all series of nanocomposites, regardless of the type of nanofillers dispersed, and as expected on the basis of percolation theory, the trend of the electrical conductivity followed a power law dependence described by the following equation:

(14) $$\sigma_{DC}=\sigma_{0}\cdot {\left(\upsilon -EPT\right)}^{t}\;\;\;\;for\;\;\nu >EPT$$

where *σ*_0_ is a pre-exponential factor that is dependent on the intrinsic electrical conductivity of the fillers, their resistance of contact and the topology of the percolation cluster [44,45], *υ* is the filler content, and *t* is a critical exponent that accounts the morphological arrangement of the filler in the percolating structure [27]. From a graphical point of view, the evolution of electrical conductivity of nanofilled composites versus filler concentration can be divided into three main phases. At the start, the conductivity, due to the small amount of additives, assumes values comparable to that of the neat polymer. Later, it begins to progressively increase, because first electrical junctions start to form, and a tunneling effect occurs between close neighbor particles. In the last phase, the increase in the filler content forms continuous paths (at percolation threshold) for the electron flow, thus enhancing the overall electrical conductivity, which in turn evolves towards a plateau value that is imposed by the tunneling resistance (*R_tunnel_*) between the filler particles, which can be evaluated according to the following expression

(15) $$R_{tunnel}=\frac{h^{2}d}{Ae^{2}\sqrt{2m\lambda }}exp^{\left(\frac{4\pi d}{h}\sqrt{2m\lambda }\right)}$$

where *h* is Plank’s constant, *A* and *d* are the surface area of the filler and the interparticle distance involved in the tunneling effect, respectively, *e* and *m* are the charge and the mass of electrons, and *λ* is the height of the barrier (generally, few eV) due to the insulating behavior of the host polymer [46,47,48].

Moreover, in the case of PLA enriched with mono-dimensional fillers, such as carbon nanotubes (N7000 and TNIMH4), the electrical conductivity already increased significantly at a filler concentration of 1.5 wt%, indicating a much lower percolation threshold (EPT) for this type of nanofiller compared to that observed for bi-dimensional ones, i.e., graphene nanoplatelets (TNIGNP, TNGNP). For these latter ones, a filler loading in the range [3÷6] wt% is required before a sharp insulator–conductor transition in the behavior of PLA is observed. With respect to hybrid systems, it is worth noting that, due to the synergistic effect between MWCNTs and GNPs, the electrical percolation threshold in such formulations was achieved with a 3 wt% total charge (with a filler weight ratio 1:1). This indicates that multiphase systems can be a valid option to improve the ability of GNP particles to easily form percolation paths, since the effectiveness of their dispersion in the polymer is improved. As a consequence, percolation is achieved with a lower filler content than that required when GNPs are used exclusively as filler for the preparation of the composites, thus enhancing their processability. Once again, the N7000 carbon nanotubes achieved the best performance (with respect to the filler TNIMH4), since better conductive networks are also established when combined with GNPs.

All of results of this electrical characterization for each of the investigated nanocomposites are collected in Table 4.

### 3.3. Thermal Conductivity of PLA-Based Nanocomposites

**Figure 11:**
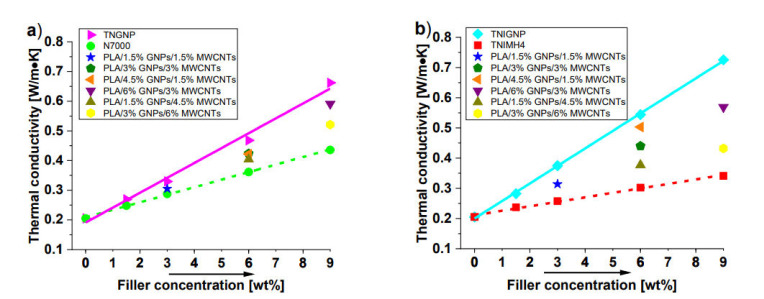
Thermal conductivity of PLA nanocomposites filled with TNGNP or N7000 and their combination in (**a**) and Table 4, and their combination in (**b**).

The thermal conductivity of a polymer can be affected by a great number of intrinsic features, and can be improved with the addition of conductive fillers into the matrix. Both MWCNTs and GNPs, given their extraordinary intrinsic thermal conductivity, are carbon-based nanofillers that are widely recognized to have a very strong influence on the thermal performance of the composites in which they are dispersed [49,50]. The thermal conductivity is shown as a function of the filler concentration up to 9 wt%, for monophase and hybrid nanocomposites realized with TNGNP and N7000 or TNIGNP and TNIMH4 carbonaceous fillers in Figure 11a,b, respectively. As expected for a pure polymer, unfilled PLA shows a low thermal conductivity value of 0.20 (W/m∙K), whereas remarkable increases are observed with the progressive addition of both types of filler, especially with addition of the graphene type. In fact, at the highest investigated filler loading (9 wt%), thermal conductivities of 0.725 (W/m∙K) and 0.662 (W/m∙K) were measured for PLA including TNIGNPs and TNGNPs, and 0.436 (W/m∙K) and 0.341 (W/m∙K) in the case of PLA filled with N7000 and TNIMH4, respectively. Therefore, a remarkable increment of the thermal conductivity of 254% with respect to that of the unfilled polymer was achieved in the best case (9 wt% TNIGNPs).

**Figure 12:**
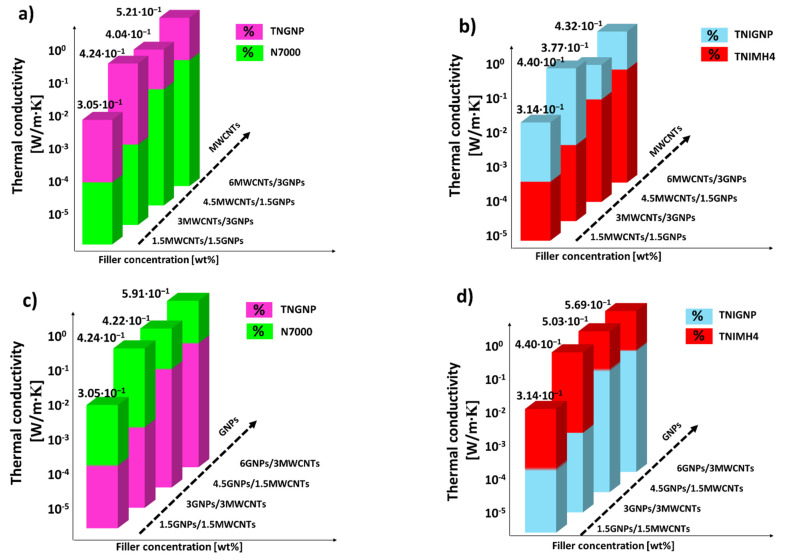
Thermal conductivity of hybrid systems including both types of filler (MWCNTs and GNPs) of type N7000 and TNGNP in (**a**,**c**) and TNIMH4 and TNIGNP in (**b**,**d**), respectively.

Intermediate values were observed for the thermal conductivities of the nanocomposites designed by combining both fillers in some selected weight ratios. In any case, such values are lower than those measured for monophase nanocomposites exclusively filled with GNP nanoparticles. For hybrid systems, as is evident from the analysis of Figure 12, the thermal conductivity is enhanced with increasing addition of graphene (regardless the type), thus confirming its key role, compared to that of carbon nanotubes, in improving the thermal transport mechanism in such nanocomposite systems.

All of the results for this thermal characterization, including thermal diffusivity values for each of the investigated nanocomposites, are reported in Table 5.

### 3.4. Design of Experiment (DoE): Dex Scatter Plot (DSP) and Main Factor Plot (MFP) for Thermal Conductivity

**Figure 13:**
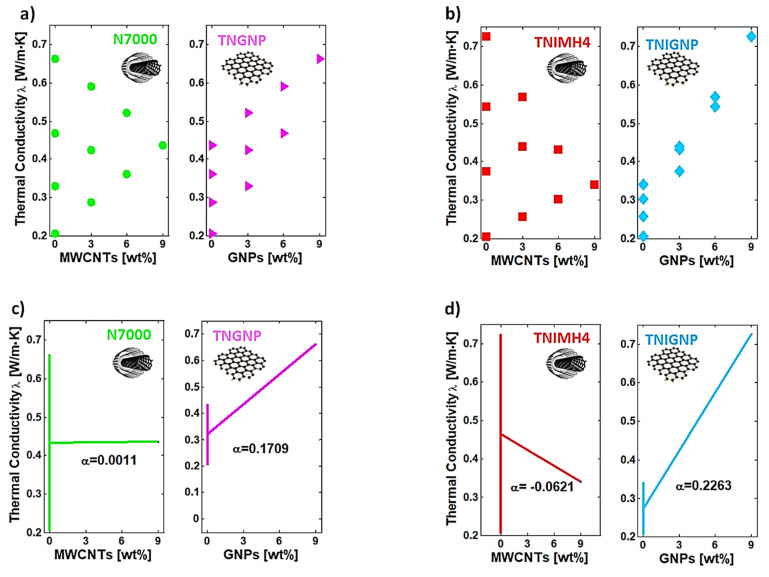
Dex Scatter Plot (DSP) and Main Factor Plot (MFP) for the experimental data of the thermal conductivity related to PLA reinforced with TNIMH4 and TNIGNP in (**a**,**b**) and with N7000 and TNGNP in (**c**,**d**), respectively.

The DoE approach leads to the Dex Scatter Plot (DSP) and Main Factor Plot (MFP), as shown in Figure 13a–d with reference to nanocomposites based on N7000 and TNGNP or TNIMH4 and TNIGNP or fillers, respectively. In brief, and for the sake of clarity, let us remember that a DSP chart shows on vertical axis the scattered experimental data of the P.F. (i.e., the thermal conductivity in the present study), which constitutes the dependent variable, while the independent variables are reported on the horizontal axis (filler loadings, ordered pairs of Equation (8)). From a graphic point of view, the DSP indicates when the P.F. reflects changes in controllable input factors, highlighting the most influential one among them, as well as providing information on its influence (improvement or worsening) [51,52]. To complement DSP information, the main factor plot (MFP) is usually reported in order to evaluate the differences between the mathematical averages for one or more input factors. From a technical point of view, it is possible to evaluate the influence of the controllable factor on the P.F. by examining the slope of the segment that joins the average points of the range of values for the performance function corresponding to the minimum and maximum levels of each factor. For a given variable, a horizontal line (parallel to the *x*-axis) indicates a null effect on the P.F., whereas the presence of a slope is indicative of a certain influence that can also be quantified and then compared to those exhibited by other input factors [53].

By analyzing these plots, it is possible to highlight the individual and combined influence of each filler on the overall thermal conductivity of the resulting nanocomposites. For formulations based on both N7000 and TNGNP or TNIMH4 and TNIGNP fillers (Figure 13a,c or Figure 13b,d), an enhancement of the thermal properties is evident due to the progressive increase in graphene loading, as demonstrated by the positive slope of the MFP segments, which show coefficients α = 0.1709 and α = 0.2263 when TNGNP and TNGINP are used as reinforcement, respectively. Due to the higher value of the α-coefficient, the latter are thus confirmed to be the best indicated for improving thermal transport in composite materials. The gradual introduction of an amount of TNHMH4 carbon nanotubes as a replacement for graphene loading led to a worsening of the thermal conductivity, as demonstrated by the negative slope of the MFP, with a coefficient α = −0.0621, whereas a sort of balancing effect (α = 0.0011) was observed when using N7000 series.

### 3.5. Response Surface Methodology (RSM)

Although introduced for first the time in the early 1950s by Box and Wilson [54], even today, Response Surface Methodology (RSM), based on Design of Experiments, represents a mathematical method for predicting the relationship between several controlled factors and experimentally observed results. Since the form of the P.F. is not known, the main aim of RSM analysis is to predict the topography of the dependent variable (response surface, R.S.) in order to identify local maxima and minima, as well as the region in which the most effective response occurs in the face of controllable input changes. In general, R.S. can be mathematically expressed as:

(16) $$R.S.=f\;\left(X_{1},\;X_{2},\;\ldots X_{n}\right)+\varepsilon$$

where *f* is the relationship between the *R.S.* and the independent input variables (*Xi*) and *ε* is the experimental error having a normal distribution with a null mean and a constant variance, as classically observed in statistical modeling. In this scenario, polynomial models are adopted for the surface prediction, and in particular, a first-order (linear) or second-order (quadratic) model, like that used in the current study, are normally sufficient for estimating the response of the most problems, especially if the problem is based on the variability of only two input variables (i.e., $wt{\%}_{MWCNTs},wt{\%}_{GNPs},\mathrm{in}\;\mathrm{our}\;\mathrm{case}$) [55,56]. From a mathematical point of view, the polynomial quadratic model (*n* = 2) can be represented by the following expression:

(17) $$R.S.=\beta_{0}+\overset{n}{\underset{i=2}{\sum }}\beta_{i}x_{i}+\overset{n}{\underset{i=2}{\sum }}\beta_{ii}x_{i}^{2}+\overset{n-1}{\underset{i=1}{\sum }}\overset{n}{\underset{j=i+1}{\sum }}\beta_{ij}x_{i}x_{j}$$

where *x_i_, x_j_* are the coded independent input variables, *β_0_* is the coefficient of intercept whereas *β_i_*, *β_ii_* and *β_ij_* are the linear, quadratic and interaction regression coefficients, respectively, which are determined by the least squares method. Here, with a particular interest in the thermal conductivity (i.e., *λ*), the aim is to derive an empirical model that correlates this property with the weight percentage of filler content, i.e., $\lambda =f\left(wt{\%}_{MWCNTs},wt{\%}_{GNPs}\right)$=$f\left(x_{1},x_{2}\right)$, for short. In accordance with Equation (17), the following quadratic polynomial approximates the values of the dependent variable λ:

(18) $$\lambda =f\left(x_{1},x_{2}\right)=\beta_{0}+\beta_{1}x_{1}+\beta_{2}x_{2}+\beta_{12}x_{1}x_{2}+\beta_{11}x_{1}^{2}+\beta_{22}x_{2}^{2}$$

**Figure 14:**
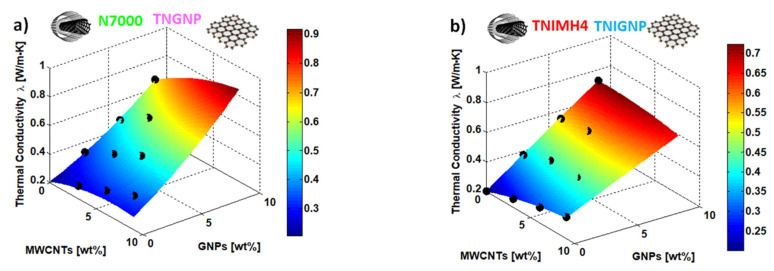
Response surface plot (full quadratic model) for the thermal conductivity depending upon the controllable input variables in the present study, i.e., the weight percentage of the fillers: N7000 and TNGNP or TNIMH4 and TNIGNP in (**a**,**b**), respectively. The black markers are the experimental data regarding thermal characterization.

The regression coefficients of the RSM are reported in Table 6, whereas a 3D plot of the R.S. with respect to the thermal conductivity of the nanocomposites including N7000 and TNGNP or TNIMH4 and TNIGNP are reported in Figure 14a,b, respectively.

It should be noted from analysis of the graph in Figure 14, that the R.S. approaches the experimental data (black markers) very well, thus evidencing the validity of the regression model for estimating the properties of a material/performance with respect to the conditioning parameters. The design of new advanced hybrid materials on the basis of simulation studies and numerical tools such as RSM may lead to the optimization of such materials without the need to test physical specimens, thus reducing their development time and avoiding the expensive costs necessitated by trial-and-error experiments. Moreover, the best composition and structure of novel materials can be achieved by coupling simulation-based approaches with statistical tools. Consequently, this will also make it possible to recognize the most sensitive controllable input factors in order to assist the design-choices in the manufacturing stage.

### 3.6. Simulation Results of Thermal Transport in Solid-State Heat Sinks

Heat sinks are passive heat exchangers designed to dissipate the heat generated by electronic devices, transferring it, by natural or forced convection, to a surrounding fluid medium (generally air), or in any case away from the device, in order to regulate the increase in temperature resulting from the operation frequency in the electronics industry. In recent years, advanced polymers have been studied for this purpose, and the advent of additive manufacturing has enabled the fabrication of 3D objects in more desirable complex shape, favoring the design of ad hoc heat sinks.

#### 3.6.1. Thermal Analysis

**Figure 15:**
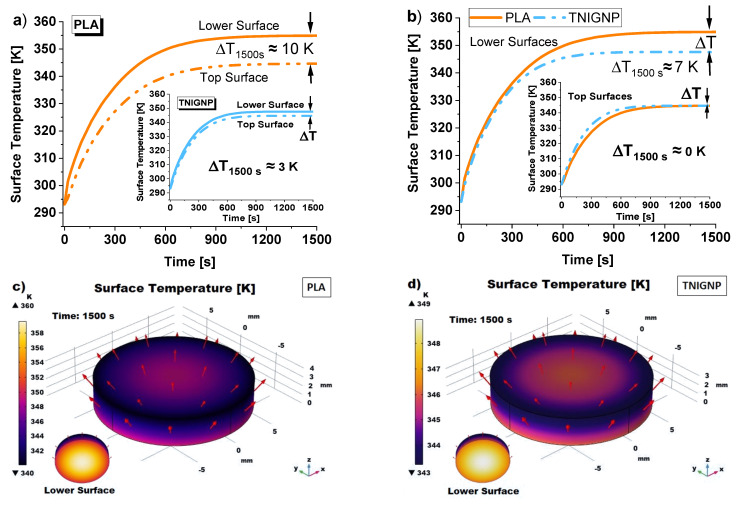
Average surface temperature profiles vs. time for heat sinks realized with (**a**) pure PLA and PLA including 9 wt% TNIGNP in the inset. A comparison of the mutual surfaces of the discs is reported in (**b**). 3D simulated views of the heat distribution on the surfaces of PLA-based disc (**c**) and on the surfaces of TNIGNP-based disc (**d**). In these last representations, red arrows indicate the direction and intensity of conductive heat flux.

Figure 15 shows a comparative analysis of the average temperature calculated on the lower and upper surfaces of the two considered heat sinks (pure PLA and PLA containing 9 wt% TNIGP), the thermal behaviors of which were simulated in the present study. It can be observed that, during the thermal transient, the temperature on each surface increases rapidly and, at the same time, the temperature difference between the lower and upper surfaces also increases; subsequently, at around 600 s, the temperature profiles tend asymptotically toward constant steady-state values, so that the temperature differences between the lower and upper surfaces also asymptotically tend toward a constant value; in fact, under steady-state conditions, the heat flow rate supplied at the lower surface is the same as that dissipated by natural convection from the upper surface and from the lateral surface.

Specifically, Figure 15a shows in the foreground the temperature profiles for both inferior and superior surfaces of the disc based on pure PLA and, in the insert of the same figure, the same profiles for that containing 9 wt% TNIGNP. The temperature difference evaluated at 1500 s between the surfaces of the individual heat sinks goes from a value of 10 K for pure PLA to 3 K for 9 wt% TNIGNP according to Equation (9), suggesting that, when subjected to the same heat flow, the higher the thermal conductivity of the solid, the lower the internal temperature gradient.

Analyzing Figure 15b, it can be observed that, under steady-state conditions at about 1500 s, the temperature difference between the lower surfaces of the two samples, through which the heat flow is supplied, remains constant at about 7 K, while the average temperatures of upper surfaces are comparable. This indicates that there is better heat transport in the graphene-based heat sink, as it is able to dissipate heat more efficiently than the disc made from unfilled polymer. The corresponding 3D temperature maps are shown in Figure 15c,d, in which the red arrows represent the direction and intensity of the conductive flux that crosses the samples under analysis and which will be analyzed in detail in the next section (Section 3.6.3. Heat Flux (Conductive/Convective) Analysis).

**Figure 16:**
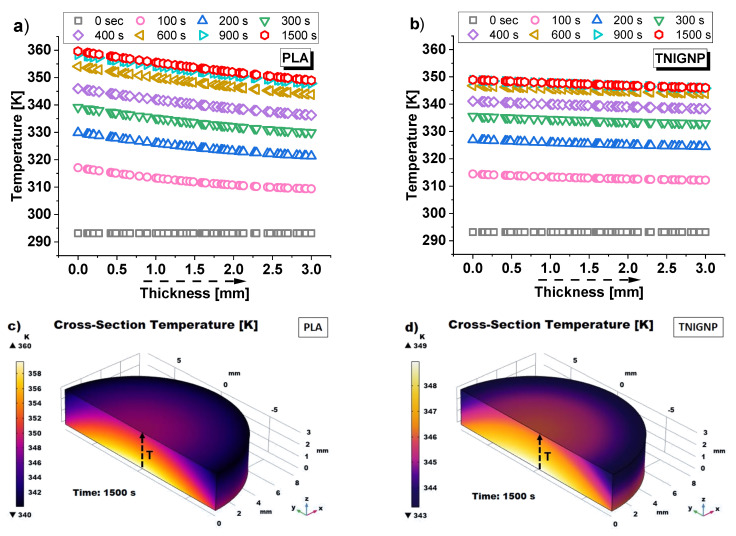
Temperature profiles (evaluated along the symmetry axis) for heat sinks made with PLA (**a**) and TNIGNP (**b**). 3D views of the cross-section temperature profiles are reported in (**c**,**d**) with an indication (black dashed arrows) of the direction in which the results were estimated.

Continuing the thermal analysis, Figure 16 presents the temperature profiles as a function of thickness in correspondence with the symmetry axis and the respective temperature maps of the simulated disk samples (PLA in Figure 16a,c and 9 wt% TNIGNP in Figure 16b,d, respectively). Starting from the initial time, *t* = 0 s, at which point the sample is at an ambient temperature of 293.15 K, following the application of heat flow, times were chosen at intervals of 100 s during the transient phase up to 400 s, and then progressively increased as steady-state conditions approach 1500 s, a time beyond which no significant temperature differences were found. With reference to the lower (*z* = 0 mm) and upper (*z* = 3 mm) surfaces, the temperature values estimated at 900 s and 1500 s and their difference for the sample of pure PLA and for that with 9 wt% TNIGNP are shown in Table 7, below.

It is worth noting that the expected drop in temperature through the thickness as the distance from the heat source increases (*z* = 0) occurs gradually for the heat sink made with TNIGNP, while it is more pronounced in that containing only PLA, as is visually evident from the temperature maps of the surfaces of the simulated heat sinks designed with PLA and TNIGNP, shown in Figure 16c,d, respectively. Once again, the thermal profile for thermal dissipative materials containing graphene-based nanoparticles is more uniform.

#### 3.6.2. Total Internal Energy Analysis

**Figure 17:**
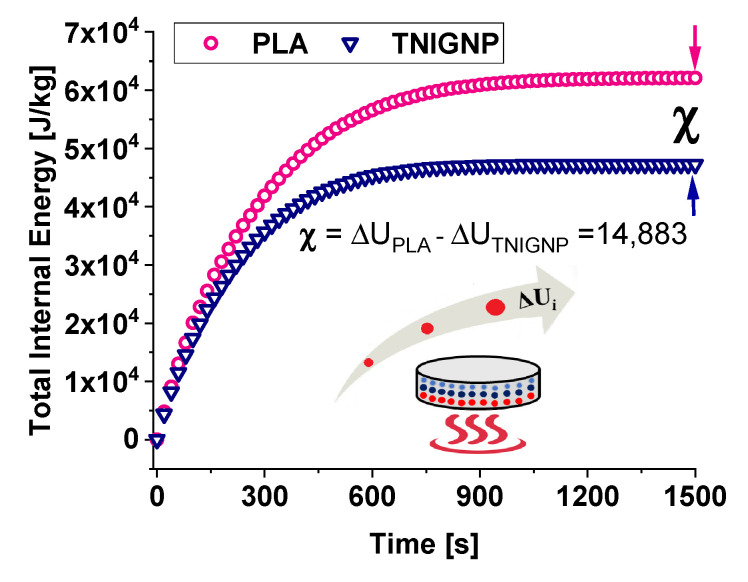
Variation of total internal energy due to the heating of the disc when it acts as a heat sink.

A comparative analysis between the thermal profiles of the two heat sink discs, each of which was stressed with the same thermal flux on the lower surface, is presented in in Figure 17, which compares the internal energy trend during heating, showing that the internal energy follows the temperature profile reported in Figure 15a. The internal energy of each sample increases progressively until it reaches its own final value under steady-state conditions at 1500 s, while the difference between the internal energy of the PLA sample and that of the 9 wt% NTIGNP sample reaches a value of approximately 14,883 J/kg.

In the case of PLA, the internal energy variation is greater than that estimated for the disc containing 9% by weight of TNIGNP due to the different level of trapped warmth, and the resulting temperature profile, within the solid.

**Figure 18:**
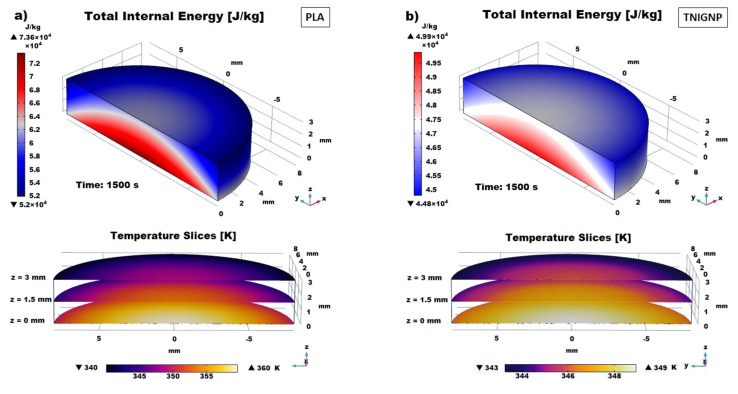
3D views of the total internal energy due to the heating of the simulated discs when they act as a heat sink: PLA in (**a**) and TNIGNP in (**b**). Temperature slices (at *z* = 0 mm, z = 1.5 mm and *z* = 3 mm) established within solid disks are also reported.

Furthermore, in Figure 18a,b, which show the 3D maps of temperature and total internal energy for PLA and TNIGNP, respectively, evaluated at *t* = 1500 s, a visual analysis of the variation along the radial direction is presented, as well as with the thickness.

#### 3.6.3. Heat Fluxes (Conductive/Convective) Analysis

**Figure 19:**
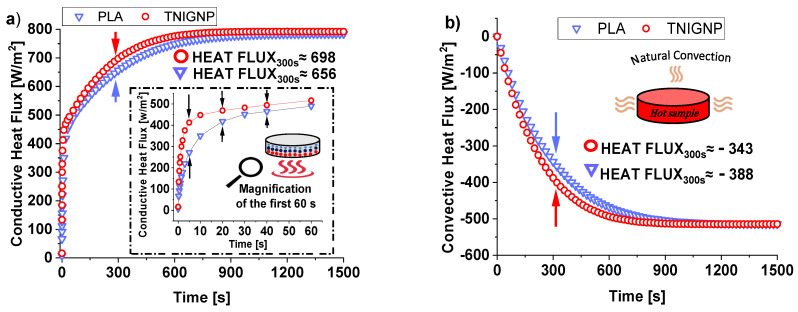
Conductive (**a**) and convective (**b**) heat flux evolution over time.

Figure 19a,b report the internal conductive heat flux trend (average values) and of the external one by natural convection as function of time, respectively. By observing them, stands out immediately that both fluxes, regardless the nature of heat sink if it is based on pure PLA or including TNIGNP, appear to be roughly equal-sided, already before steady-state condition. At the end of observing time of 1500 s a value of 784 (W/m^2^) and 792 (W/m^2^) are estimated for the conductive heat flux whereas 514.4 (W/m^2^) and 514.1 (W/m^2^) are the values for the convective flux of PLA and TNIGNP, respectively. On the other hand, as clearly highlighted in the magnification of the first 60 s depicted in the inset of Figure 19a, significant changes can be observed during the transient phase, especially for conductive heat flux. This difference is due to the graphene particles, which, as already explained, favor thermal transport when introduced in the polymer matrix by reducing the temperature gradient proportionally, by means of thermal conductivity. When thermal equilibrium is reached, the conductive heat flux will have been completely transferred to the surrounding environment, and there will be no further changes with respect to the thermal properties.

In light of this, the moment of time at 300 s, which falls approximately in the middle of the transient phase, is taken as a reference for the performance comparison with respect to the heat fluxes of the two simulated disks. At this selected time, a difference of about 42 (W/m^2^) and 45 (W/m^2^) is estimated for the conductive and convective flux, respectively. In steady-state condition (1500 s) the conductive and convective fluxes stabilize to a value of 701.29 (W/m^2^) and 705.61 (W/m^2^) or −514.04 (W/m^2^) and −514.49 (W/m^2^) for PLA and TNIGNP heat sink, respectively.

**Figure 20:**
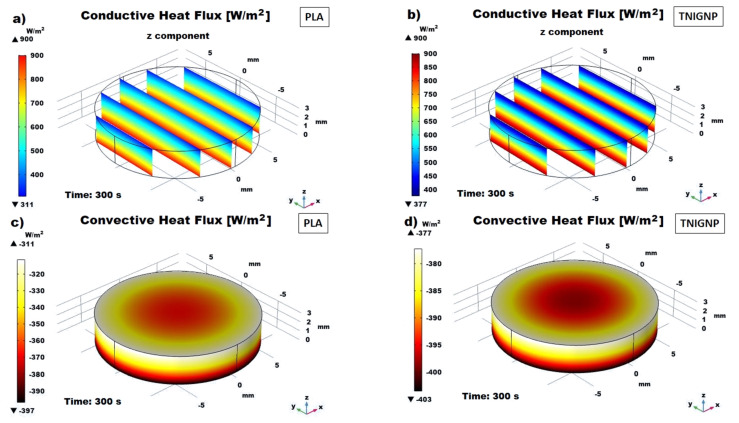
3D views of the conductive heat flux in some cross-sections of PLA (**a**) and TNIGNP (**b**); their corresponding convective heat flux is presented in (**c**,**d**), respectively.

3D views of their distribution profiles within the solid are shown in Figure 20. More specifically, Figure 20a,b respectively report the conductive heat flux (z component) for PLA and TNIGNP along some selected equidistant cross-sections of the materials. Consulting such graphics with their relative color bars not only helps to quantify these fluxes, but also to discriminate the different rates (greater for PLA containing TNIGNP than for pure PLA). Finally, Figure 20c,d illustrate the profile of convective heat flux at the solid/air exchange surface for pure and filled polymer. It is worth noting that, in both cases, the convective flux is greater in the central part of the solids, due to both the greater exchange surface compared to the side walls as well as the higher temperature achieved in this region, in line with the thermal profiles previously discussed.

## 4. Discussion

The thermal behavior of poly(lactic acid) (PLA) filled with different carbonaceous fillers was experimentally, theoretical and numerically investigated.

Nanocomposites based on N7000-type CNTs, despite their small A.R. of 150, showed better thermal conductivity than those realized with TNIMH4. This result may be attributed to their shorter length, which leads to their easy and better dispersion, which in turn results in better interparticle contacts in the percolating network. The TNIMH4 nanotubes show an A.R. of 1000, which, combined with the longer length, most likely prevents good dispersion in the melt during the extrusion process. Of course, this different dispersion state conditions the thermal properties of the resulting composites.

The best thermal performances were revealed for composites including graphene-based particles, rather than carbon nanotubes. The explanation for this is to be found in the different interface interactions between the organic polymer and the carbonaceous fillers. For the 1-dimensional ones, like nanotubes, the Kapitza resistance (*Rk*) shows higher values due to the inner surfaces that are poorly wetted by the PLA in contrast to what happens for graphene nanoplatelets, where these surface impregnation phenomena are favored by their planar shapes. In this last case, a lowering of the differences of phonon density of the states between the constituent phases determines a more effective phononic heat flow.

Despite the high intrinsic thermal conductivities of both fillers (as reported in Table 1), the overall thermal performances of the resulting nanocomposites were decisively far from these values. The reason for this is the fact that, in percolated structures, macroscale properties are strongly conditioned by a great number of factors that occur at the micro- and nanoscale level, such as aggregations and dispersion states, functional group effects, crystallinity and surface tensions of the matrix, and so on. All these effects are not yet fully understood or predictable in the design stage, and therefore, further studies are needed in order to add knowledge in the field and to obtain new findings. Theoretical and simulation studies can support experimental research in order to achieve this objective. Design of Experiment (DoE), combined with Main Factor Plot (MFP) and the Response Surface Methodology (RSM) can be successfully applied in many experimental situations, and more recently, they have increasingly been used in the field of the design and development of materials. In this context, these statistical approaches appear to be particularly useful for experiments, especially when they are based on destructive tests, which lead to the depletion of resources, or when they are based on rare/expensive materials, as in the case of carbon-based fillers. These techniques improve experimental efficiency, as well as being able to assist the designer in comprehending factor interactions, since they provide detailed information with respect to process evolution. Additionally, their use allows us to achieve new insights into the behavior of advanced nanocomposites. The DoE approach employed in this study made it possible to quantify the influence of each of the investigated fillers, while RSM allowed us to derive a polynomial equation that was able to correlate their loading levels with the thermal conductivity. Given the reliability of the predictive models, whose results match very well with the experimental data, the theoretical and numerical studies are able to provide useful information during the design stage of nanocomposites by limiting the classical experimental activity based on onerous trial and error approaches, in which something is tested until one finds the most successful parameters. Multiphysics simulations make it possible to investigate the thermal behavior of polymer-based nanocomposites for their potential use in heat transfer applications. In the present study, by comparing the thermal performances of two heat sinks, based on unfilled PLA and reinforcement with 9 wt% TNIGNP, it was proved that the introduction of filler significantly improved the thermal properties. Therefore, simulation results also confirmed the key role of graphene in improving the thermal features of materials in which it is dispersed. Moreover, thanks to the thermal simulation studies, the working temperatures of the designed heat sinks can be tailored to be lower than the glass transition, thus avoiding the risk of material degradation in the case of their use in heat transfer applications.

## 5. Conclusions

This paper mainly addressed the study of the thermal behavior of poly(lactic acid) composites reinforced with different carbonaceous fillers produced via melt compounding. An experimental characterization in terms of morphology, electrical and thermal properties was carried out, and the results were discussed with reference to theoretical expectations. Carbon nanotubes, especially of type N7000, were found to be the best performing in terms of electrical properties, given their greater efficiency in creating percolation paths within the polymer. The percolation threshold was achieved with a filler concentration lower than 1.5 wt%, whereas a remarkable electrical conductivity of 2 S/m was measured at the highest investigated loading. For nanocomposites based on both types of graphene, the EPT fell in a wider range of [3÷6] wt%, and an electrical conductivity value of about 7 × 10^−2^ S/m was achieved in the best case (9 wt% TNIGNP). In contrast, industrial graphene nanoplatelets (TNIGNP) were found to be the best performing from a thermal point of view, since an increment of the thermal conductivity by 254% with respect to that of unfilled polymer was achieved in the best case (at concentration of 9 wt%). By using MWCNTs, the improvement in thermal conductivity with respect to the pure PLA was reduced to the still-interesting value of 112%, due to the conductivity value of 0.436 W/m∙K measured for nanocomposites including 9 wt% N7000. Design of Experiment, including the response surface methodology, was performed to introduce predictive numerical models that would be useful for the design of such advanced materials. With reference to the best thermally performing nanocomposite (TNIGNP) and unfilled PLA, multiphysics simulations were carried out to numerically investigate and compare their thermal behavior when used as heat sinks for potential heat-transfer applications. A lower surface heating and a lower achieved temperature were observed at equilibrium (about 354 K and 344 K, for neat PLA and PLA with TNIGNPs), as well as a more efficient environmental heat exchange, especially during the transient phase, with a difference of 45 [W/m^2^] at an observation time of 300 s for the two simulated heat sinks. At the same time, a greater heat convective flux (642 W/m^2^) was exhibited by the TNIGNP-based heat sink with respect to that realized with pure PLA (613 W/m^2^), which is an indicator of better thermal transport.

## Figures and Tables

**Table 1:** Thermal conductivity (at room temperature) of some well-known polymers for AM and of some dispersive fillers classically adopted for improving their thermal behavior [24].

**Polymer**		
**Polymer Name**	**Acronym**	**Thermal Conductivity [W/m∙K]**
High density polyethylene	HDPE	0.44
Polyphenylsulfone	PPSU	0.35
Ethylene Vinyl Acetate	EVA	0.34
Acrylonitrile Butadiene Styrene	ABS	0.33
Poly(Butylene Terephthalate)	PBT	0.29
Nylon-6	PA6	0.25
Polyether Ether Ketone	PEEK	0.25
Poly(dimethylsiloxane)	PDMS	0.25
* Poly(lactic) Acid	PLA	0.20
Polymethylmethacrylate	PMMA	0.21
Polyvinyl Chloride	PVC	0.19
Polyvinylidene Difluoride	PVDF	0.19
Polystyrene	PS	0.14
Polypropylene	PP	0.12
**Filler**		
**Group**	**Type**	**Thermal Conductivity [W/m∙K]**
Carbon-based Fillers	Carbon nanotubes	2000÷6000 (longitudinal)
Carbon-based Fillers	Graphite	100÷400 (in plane)
Carbon-based Fillers	Carbon Black (CB)	6÷174
Metallic Fillers	Copper (Cu)	483
Metallic Fillers	Silver (Ag)	450
Metallic Fillers	Gold (Au)	345
Metallic Fillers	Aluminum (Al)	204
Ceramic Fillers	Boron nitride (BN)	250÷300
Ceramic Fillers	Aluminum oxide (Al_2_O_3_)	30

* Value from the experimental characterization carried out in the present study.

**Table 2:** List of mono-filler and bi-filler composites on the base of PLA, industrial graphene (TNIGNP) and industrial MWCNTs (TNIMH4) as well as graphene TNGNP and MWCNT of type N7000.

Composition	GNP Content [wt%]	MWCNT Content [wt%]	PLA Content [wt%]	GNP/MWCNT [Ratio]
PLA	-	-	100	-
Mono-phase nanocomposites based on GNPs (TNIGNP or TNGNP)				
PLA/1.5% GNP	1.5	-	98.5	-
PLA/3% GNP	3	-	97	-
PLA/6% GNP	6	-	94	-
PLA/9% GNP	9	-	91	-
Mono-phase nanocomposites based on MWCNTs (TNIMH4 or N7000)				
PLA/1.5% MWCNT	-	1.5	98.5	-
PLA/3% MWCNT	-	3	97	-
PLA/6% MWCNT	-	6	94	-
PLA/9% MWCNT	-	9	91	-
Multiphase (hybrid) nanocomposites based on GNPs/MWCNTs (TNIGNP/TNIMH4 or TNGNP/N7000)				
PLA/1.5% GNP/1.5% MWCNT	1.5	1.5	97	1:1
PLA/3% GNP/3% MWCNT	3	3	94	1:1
PLA/4.5% GNP/1.5% MWCNT	4.5	1.5	94	3:1
PLA/6% GNP/3% MWCNT	6	3	91	2:1
PLA/1.5% GNP/4.5% MWCNT	1.5	4.5	94	1:3
PLA/3% GNP/6% MWCNT	3	6	91	1:2

**Table 3:** Conditions for the solving the thermal energy equation.

Initial and Boundary Condition	Equation	Validity
*t* = 0	*T* = *T*_0_	∀r,∀z
r=0	$\frac{\partial T}{\partial r}=0$	∀z,∀t>0
r=R	$-K\frac{\partial T}{\partial r}=h\left(T-T_{\infty }\right)$	∀z,∀t>0
z=0	$-K\frac{\partial T}{\partial r}=q$	∀r,∀t>0
z=H	$-K\frac{\partial T}{\partial r}=h\left(T-T_{\infty }\right)$	∀r,∀t>0

**Table 4:** Results of the electrical characterization.

FILLER:	TNIGNP (GNP), TNIMH4 (MWCNT)		TNGNP (GNP), N7000 (MWCNT)	
Composites	Electrical Conductivity [S/m]		Electrical Conductivity [S/m]	
PLA	5.9 × 10^−10^	(±1.30 × 10^−11^)	5.9 × 10^−10^	(±1.30 × 10^−11^)
1.5%GNP	7.8 × 10^−10^	(±1.82 × 10^−11^)	1.3 × 10^−9^	(±6.78 × 10^−10^)
3%GNP	1.6 × 10^−9^	(±1.5 × 10^−10^)	2.8 × 10^−9^	(±4.60 × 10^−10^)
6%GNP	0.0133	(±2.12 × 10^−3^)	6.04 × 10^−6^	(±1.13 × 10^−7^)
9%GNP	0.07932	(±5.88 × 10^−3^)	2.7 × 10^−5^	(±2.01 × 10^−6^)
1.5%MWCNT	1.2 × 10^−5^	(±5.94 × 10^−6^)	0.17	(±2.50 × 10^−2^)
3%MWCNT	0.0121	(±2.74 × 10^−3^)	0.69	(±2.73 × 10^−2^)
6%MWCNT	0.0783	(±1.01 × 10^−3^)	0.89	(±2.63 × 10^−2^)
9%MWCNT	0.26404	(±1.81 × 10^−2^)	2.00	(±1.21 × 10^−2^)
1.5%GNP + 1.5%MWCNT	2.41 × 10^−5^	(±2.21 × 10^−6^)	0.24207	(±9.98 × 10^−2^)
1.5%GNP + 4.5%MWCNT	0.11393	(±1.31 × 10^−2^)	0.21099	(±4.32 × 10^−2^)
3%GNP + 3%MWCNT	0.13928	(±1.44 × 10^−2^)	0.67	(±5.34 × 10^−2^)
4.5%GNP + 1.5%MWCNT	0.03153	(±1.60 × 10^−2^)	0.30336	(±1.19 × 10^−2^)
3%GNP + 6%MWCNT	0.19565	(±2.53 × 10^−2^)	0.58949	(±1.55 × 10^−2^)
6%GNP + 3%MWCNT	0.13234	(±2.47 × 10^−2^)	0.30879	(±6.19 × 10^−2^)

**Table 5:** Thermal results for each formulation investigated in the present study.

**PLA 3D850 (Polymer), TNIGNP (GNPs), TNIMH4 (MWCNTs)**				
**Composites**	**Thermal Conductivity [W/m∙K]**		**Mean Thermal Diffusivity [mm^2^/s]**	
PLA	2.05 × 10^−1^	(±3.91 × 10^−3^)	0,15 × 10^−1^	(±6.15 × 10^−2^)
1.5%GNP	2.82 × 10^−1^	(±7.21 × 10^−4^)	2.10 × 10^−1^	(±1.18 × 10^−2^)
3%GNP	3.75 × 10^−1^	(±4.11 × 10^−3^)	3.94 × 10^−1^	(±1.78 × 10^−2^)
6%GNP	5.44 × 10^−1^	(±7.41 × 10^−3^)	4.34 × 10^−1^	(±1.96 × 10^−2^)
9%GNP	7.25 × 10^−1^	(±4.24 × 10^−2^)	6.29 × 10^−1^	(±6.15 × 10^−3^)
1.5%MWCNT	2.37 × 10^−1^	(±1.27 × 10^−3^)	1.75 × 10^−1^	(±4.01 × 10^−3^)
3%MWCNT	2.58 × 10^−1^	(±2.85 × 10^−3^)	1.88 × 10^−1^	(±4.86 × 10^−3^)
6%MWCNT	3.02 × 10^−1^	(±2.40 × 10^−3^)	2.29 × 10^−1^	(±3.52 × 10^−3^)
9%MWCNT	3.41 × 10^−1^	(±2.95 × 10^−3^)	2.41 × 10^−1^	(±3.06 × 10^−3^)
1.5%GNP + 1.5%MWCNT	3.14 × 10^−1^	(±3.94 × 10^−3^)	2.33 × 10^−1^	(±8.06 × 10^−3^)
1.5%GNP + 4.5%MWCNT	3.77 × 10^−1^	(±1.03 × 10^−2^)	2.99 × 10^−1^	(±5.81 × 10^−3^)
3%GNP + 3%MWCNT	4.40 × 10^−1^	(±1.20 × 10^−2^)	3.61 × 10^−1^	(±2.08 × 10^−2^)
4.5%GNP + 1.5%MWCNT	5.03 × 10^−1^	(±4.88 × 10^−3^)	3.91 × 10^−1^	(±2.93 × 10^−2^)
3%GNP + 6%MWCNT	4.32 × 10^−1^	(±8.34 × 10^−3^)	3.57 × 10^−1^	(±7.88 × 10^−3^)
6%GNP+ 3%MWCNT	5.69 × 10^−1^	(±1.72 × 10^−2^)	4.86 × 10^−1^	(±8.58 × 10^−3^)
**PLA 3D850 (Polymer), TNGNP (GNPs), N7000 (MWCNTs)**				
**Composites**	**Thermal Conductivity [W/m∙K]**		**Mean Thermal Diffusivity [mm^2^/s]**	
PLA	2.05 × 10^−1^	(±3.91 × 10^−3^)	0.15 × 10^−1^	(±6.15 × 10^−2^)
1.5%GNP	2.69 × 10^−1^	(±2.31 × 10^−3^)	2.08 × 10^−1^	(±7.18 × 10^−3^)
3%GNP	3.30 × 10^−1^	(±5.08 × 10^−3^)	3.35 × 10^−1^	(±9.07 × 10^−2^)
6%GNP	4.68 × 10^−1^	(±1.50 × 10^−2^)	5.08 × 10^−1^	(±2.50 × 10^−2^)
9%GNP	6.62 × 10^−1^	(±1.85 × 10^−2^)	6.24 × 10^−1^	(±5.72 × 10^−2^)
1.5%MWCNT	2.47 × 10^−1^	(±2.44 × 10^−2^)	2.57 × 10^−1^	(±1.15 × 10^−2^)
3%MWCNT	2.88 × 10^−1^	(±1.81 × 10^−2^)	2.90 × 10^−1^	(±6.45 × 10^−2^)
6%MWCNT	3.61 × 10^−1^	(±8.62 × 10^−3^)	2.64 × 10^−1^	(±6.12 × 10^−3^)
9%MWCNT	4.36 × 10^−1^	(±7.83 × 10^−3^)	3.44 × 10^−1^	(±2.17 × 10^−2^)
1.5%GNP + 1.5%MWCNT	3.05 × 10^−1^	(±6.47 × 10^−3^)	2.24 × 10^−1^	(±3.25 × 10^−3^)
1.5%GNP + 4.5%MWCNT	4.04 × 10^−1^	(±1.68 × 10^−2^)	2.97 × 10^−1^	(±1.28 × 10^−2^)
3%GNP + 3%MWCNT	4.24 × 10^−1^	(±8.60 × 10^−3^)	3.28 × 10^−1^	(±6.19 × 10^−3^)
4.5%GNP + 1.5%MWCNT	4.22 × 10^−1^	(±2.18 × 10^−2^)	5.15 × 10^−1^	(±7.26 × 10^−2^)
3%GNP + 6%MWCNT	5.21 × 10^−1^	(±1.67 × 10^−2^)	4.21 × 10^−1^	(±2.60 × 10^−2^)
6%GNP + 3%MWCNT	5.91 × 10^−1^	(±1.88 × 10^−2^)	4.96 × 10^−1^	(±4.48 × 10^−2^)

**Table 6:** RSM regression coefficients for the quadratic response of the thermal conductivity λ.

Coefficient	β_0_	β_1_	β_2_	β_12_	β_11_	β_22_
Value for λ: (N7000-TNGNP):	+0.2073	+0.0270	+0.0331	+0.0022	−1.7143 × 10^−4^	+0.0019
Value for λ (TNIMH4-TNIGNP):	+0.2021	+0.0231	+0.0591	−0.0023	−9.0714 × 10^−4^	−1.4286 × 10^−4^

**Table 7:** Lower/upper surface temperatures at 900 s and 1500 s.

Heat Sink	Lower/Upper Surface	T (900 s) [K]	T (1500 s) [K]	ΔT [K]
PLA	*z* = 0 mm	358.4	359.6	1.2
	*z* = 3 mm	347.8	349.0	0.1
TNIGNP	*z* = 0 mm	348.8	348.9	0.1
	*z* = 3 mm	345.8	345.9	0.1

## Data Availability

Data are contained within the article.
